# Ileal Inflammation May Trigger the Development of GP2-Specific Pancreatic Autoantibodies in Patients with Crohn's Disease

**DOI:** 10.1155/2012/640835

**Published:** 2012-10-17

**Authors:** Polychronis Pavlidis, Ourania Romanidou, Dirk Roggenbuck, Maria G. Mytilinaiou, Faris Al-Sulttan, Christos Liaskos, Daniel S. Smyk, Andreas L. Koutsoumpas, Eirini I. Rigopoulou, Karsten Conrad, Alastair Forbes, Dimitrios P. Bogdanos

**Affiliations:** ^1^Division of Transplantation Immunology and Mucosal Biology, King's College London School of Medicine at King's College Hospital, London SE5 9RJ, UK; ^2^Department of Gastroenterology and Clinical Nutrition, University College Hospital, 250 Euston Road, London NW1 2PG, UK; ^3^Cellular Immunotherapy and Molecular Immunodiagnostics, Center for Research and Technology, Thessaly, 41222 Larissa, Greece; ^4^Faculty of Natural Science, Lausitz University of Applied Sciences, 01968 Senftenberg, Germany; ^5^GA Generic Assays GmbH, L.-Erhard-Ring 3, Dahlewitz, 15827 Berlin, Germany; ^6^Department of Medicine, University of Thessaly Medical School, Viopolis, Larissa 41110, Greece; ^7^Institute of Immunology, Technical University Dresden, Fetscherstrasse 74, 01307 Dresden, Germany

## Abstract

Why zymogen glycoprotein 2 (GP2), the Crohn's disease (CD)-specific pancreatic autoantigen, is the major target of humoral autoimmunity in inflammatory bowel diseases (IBD) is uknown. Recent evidence demonstrates that GP2 is also present on the apical surface of microfold (M) intestinal cells. As the colon lacks GP2-rich M cells, we assumed that patients with colonic CD are seronegative for anti-GP2. Anti-GP2 antibodies were tested in 225 CDs, including 45 patients with colonic location (L2), 45 with terminal ileum (L1) and 135 with ileocolonic involvement; 225 patients with ulcerative colitis (UC) were also tested. Anti-GP2 reactivity was detected in 59 (26.2%) CDs and 15 (6.7%) UCs (*P* < 0.001). Only 5 CDs with L2 had anti-GP2 antibodies, compared to 54/180 (30.0%, *P* = 0.0128) of the CDs with L1 and L3. Anti-GP2 antibody positive CD patients had higher ASCA titres compared to seronegative cases. Amongst the 128 CD patients with previous surgical intervention, 45 (35.0%) were anti-GP2 antibody positive compared to 14/97 (14.0%) without surgical (*P* < 0.001). Our data support the assumption that ileal inflammation is required for the development of anti-GP2 antibodies in CD, and suggest that the intestine rather than the pancreatic juice is the antigenic source required for the initiation of anti-GP2 antibodies.

## 1. Introduction

Pancreatic autoantibodies (PAB) detected by indirect immunofluorescence (IIF) are specific markers of Crohn's disease (CD), being present in approximately 27–39% of patients with this condition, but in fewer than 8% of patients with ulcerative colitis (UC) or other disorders unrelated to inflammatory bowel diseases (IBD) [1–7]. The major target antigen of PAB has recently been elucidated as a pancreatic glycosyl phosphoinositol (GPI) membrane-anchored protein, also known as zymogen glycoprotein 2 (GP2) [8].

It was previously believed that GP2 was exclusively expressed by pancreatic acinar cells [9, 10], but recent studies have clearly demonstrated that GP2 is also located in the microfold (M) cells of the follicle-associated epithelium (FAE) of intestinal Peyer's patches [11]. Thus, it appears that GP2 is located in the intestine, as well as the exocrine pancreas, and this may explain its intriguing autoantigenicity in patients with CD [9–13]. 

Direct proof of the relationship between the autoantigenicity of GP2 and its peculiar location on the apical surface of the GP2-rich intestinal M cells has not yet been obtained [12]. PCR analysis of colonic biopsy material of anti-GP2 antibody positive patients with CD suggested that there is a CD-specific overexpression of GP2 in this disease [8], but the data are scarce and far from conclusive [12]. 

While M cells are found in abundance in the small intestine and in particular in the ileum, they are hardly detectable in the large intestine [14]. We assumed that the production of GP2 autoantibodies is triggered during ileal inflammation and that high expression of GP2 by M cells in the inflamed ileal environment is important for the release of this antigen and its continual exposure to the immune system [12]. If this holds true, it would be expected that patients with exclusively colonic CD would lack anti-GP2 antibodies as compared to patients with ileal or ileocolonic inflammation. Such information would also provide clues as to whether GP2 autoantibodies participate in the immunopathogenicity of CD or are just epiphenomena, secondary to ileal inflammation.

## 2. Patients and Methods

### 2.1. Patients

Serum samples of 450 patients from a cohort of 854 follow-up IBD patients seen in the outpatient clinics of one of the authors (A. Forbes) who runs a tertiary referral service in the UK (currently at University College Hospital, London) were tested. 

The study population included 225 patients with CD (men/women: 98/127, 36.0 ± 14.3 years; disease duration 13.0 ± 10.1 years) and 225 UC patients (male/female: 113/112; age median: 51.0 ± 15.7; disease duration median: 14.0 ± 12.9, Table 1).

The diagnoses of CD and UC were based on current standard clinical, radiological, endoscopic, and histological criteria (Lennard-Jones criteria) [15]. The disease phenotype was determined based on the Montreal classification [16]. 

Disease location was the criterion for the selection of CD patients. All the patients with ileal (L1 = 45) and colonic (L2 = 45) involvement were included. A proportionally larger group of patients with extensive disease (ileal and colonic involvement, L3 = 135) was selected reflecting the higher prevalence in the original population. An equal number of patients with UC were randomly selected.

Follow-up samples were taken from 40 opportunistically selected patients (CD: 20, UC: 20) at various time points (median CD follow up of 3.0 ± 1.3 years; median UC follow up: 3.0 ± 1.0 years).

In addition, 75 serum samples from 50 healthy blood donors and 25 patients with irritable bowel syndrome have been included as normal and pathological controls, respectively. Laboratory, histological and clinical data recorded in an electronic database were used to analyse patients stratified into groups according to the presence or not of anti-GP2 antibodies.

The study was conducted in accordance with the Helsinki declaration and approved by the local ethics committees. Written informed consent was obtained from each individual. All sera had been stored at −20°C before analysis.

#### 2.1.1. Detection of Anti-GP2 Antibodies by ELISA

IgG anti-GP2 antibodies were tested in serum samples of patients with IBD by a commercial ELISA (Generic Assays, Dahlewitz/Berlin, Germany) [17], according to the manufacturer's instructions. The assay is based on recombinant human GP2 expressed in *Spodoptera frugiperda* 9 cells as solid-phase antigen, as described previously [18]. Briefly, the plasmid pcDNA3.1 + GP2-trunc-Thrombin-His was used which codes the amino acid sequence of GP2 isoform BAA88166 (pancreatic GP2 alpha form) corresponding to the formal isoform 2 (NP_001493) missing the last 8 amino acids at the N-terminal end [19]. The cutoff for positivity was set to 20 AU/mL, as recommended by the manufacturer. The anti-GP2 IgG ELISA displayed an intra-assay variability of 4.3% and an interassay variability of 5.6% for samples giving 29 AU/mL and 27 AU/mL, respectively.

#### 2.1.2. Detection of Antibodies to *Saccharomyces cerevisiae *(ASCA) by ELISA

In view of the high specificity of ASCA antibodies for CD and the frequent cooccurrence with pancreatic autoantibodies described in previous studies [8, 20], patients' serum samples were also tested for ASCA antibody reactivity. A commercially available ELISA (INOVA Diagnostics) kindly provided by Dr. Gary L. Norman was used for the quantitative determination of IgA and IgG ASCA antibodies, following the manufacturer's protocol. A cutoff for positivity was set to 25 AU/mL, as recommended by the manufacturer. The intra-assay coefficient of variation was 3.7% for a sample containing 65 AU/mL of ASCA IgA and 4.5% for a sample containing 45 AU/mL of ASCA IgG. The inter-assay coefficient of variation was 4.5% for a sample containing 52 AU/mL and 2.5% for a sample containing 52 AU/mL of ASCA IgA and ASCA IgG, respectively. 

### 2.2. Statistics

All statistical tests were performed using the SPSS 15.0 statistical software package (SPSS Inc., Chicago, Illinois, USA). Prizm software (by GraphPad Software Inc., La Jolla, California, USA) was used for drawing the presented figures. An assumption of nonparametric variables was made and the comparisons were performed with Mann-Whitney, Fisher exact, and chi-square tests as appropriate. Wherever required a non-parametric Spearman correlation was performed. Results are presented as percentages and medians with standard deviation error and odd ratios with 95% confidence intervals (CI). All *P* values reported are for two-tailed analysis.

## 3. Results

### 3.1. IgG Anti-GP2 Antibodies in CD, UC, and Non-IBD Controls

**Figure 1:**
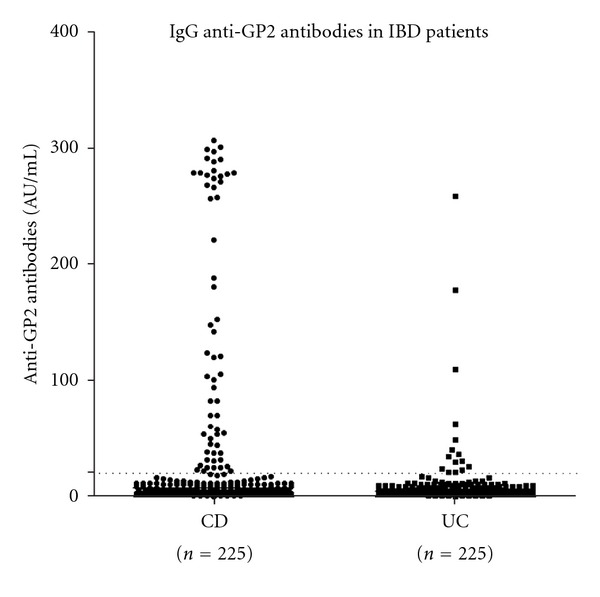
IgG anti-GP2 antibodies in 225 patients with Crohn's disease (CD) and 225 patients with ulcerative colitis (UC). A cutoff of 20 AU/mL established by the manufacturer of the commercial ELISA (Generic Assays) is indicated with a dot line.

IgG anti-GP2 reactivity was detected in 59 (26.2%) patients with CD and 15 (6.7%) patients with UC (*χ* ^2^ = 31.3, df = 1, *P* < 0.000, odds ratio: 4.98, 95% CI 2.73 to 9.08). The titres were significantly higher in CD in comparison to UC patients (*U*: 18920, *P* < 0.0001, Figure 1).

IgG antibodies were present in one (1/75, 1.33%; 47 AU/mL) non-IBD control tested (a 37-year-old female suffering from irritable bowel syndrome without family history of IBD).

The sensitivity of IgG anti-GP2 antibodies for IBD versus non-IBD controls was 16%, the specificity 99%, and the likehood ratio 12.33. When comparing CD versus UC then the sensitivity was 26%, the specificity 93%, and the likehood ratio 3.93.

### 3.2. IgG Anti-GP2 Antibodies in CD Patients according to Disease Location

**Figure 2:**
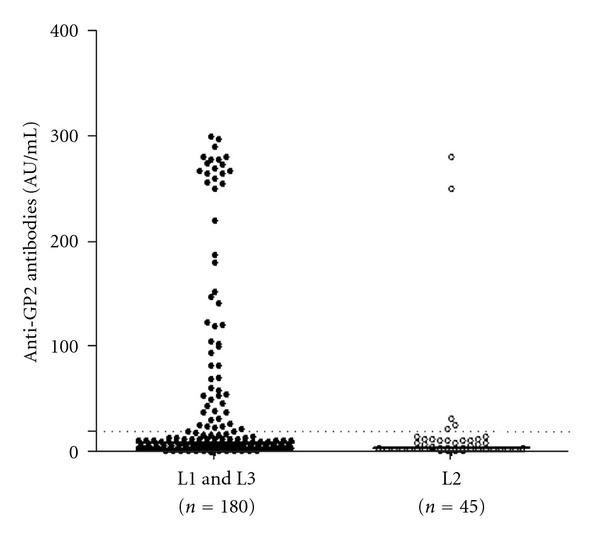
Anti-GP2 antibody titres in 225 patients with Crohn's disease (CD) stratified in two groups: patients with restricted colonic location (L2) and patients with ileal (ileal or ileocolonic) location (L1 and L3), according to Montreal classification.

CD patients with ileal (L1) or extensive disease (L3) presented higher prevalence of anti-GP2 IgG (*P* = 0.0128) with significantly higher titres as shown in Figure 2. Thus, anti-GP2 antibodies were present in 5/45 (11.1%) L2 CD, representing just 8.5% (5/59) of the total anti-GP2 positive CD cohort and 2.2% (5/225) of the total CD population included in the present study. This was statistically less prevalent compared to the 30% (54/180) anti-GP2 seropositivity seen in patients with L1 and L3, who represent the 91.5% of the total anti-GP2 seropositive CD patients and 24% (54/225) of the total CD population.

### 3.3. IgG Anti-GP2 Antibodies versus ASCA (IgA and IgG)

**Figure 3:**
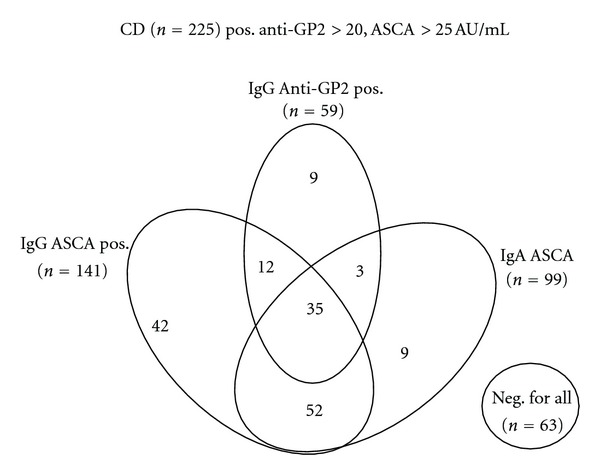
Venn Diagram showing IgA ASCA, IgG ASCA, and IgG anti-GP2 antibody reactivity of the 225 Crohn's disease (CD) patients.

A summary of the results is given as a Venn diagram in Figure 3. Amongst the 225 patients with CD, 141 (62.7%) and 99 (44.0%) had IgG and IgA ASCA, respectively. Overall, 153 (68.0%) CD patients had IgG and/or IgA ASCA compared to 28 (12.4%) UC patients (*P* < 0.0001). Among the ASCA (IgA and/or IgG) positive CD and UC patients, 50 (33%) and 5 (18%) were positive for IgG anti-GP2, respectively. Overall, 57 (40%) of the IgG and 38 (38%) of the IgA ASCA positive patients had anti-GP2 antibody reactivity, respectively. Only 35 (15.6% of the total 225) CD patients had simultaneous reactivity for ASCA (both IgG and IgA) and anti-GP2. These represented 59.3% of the total (*n* = 59) anti-GP2 antibody reactive cases. Among the 62 (28.0%) ASCA seronegative CD patients, 9 (15.0%) were positive for anti-GP2 IgG.

**Figure 4:**
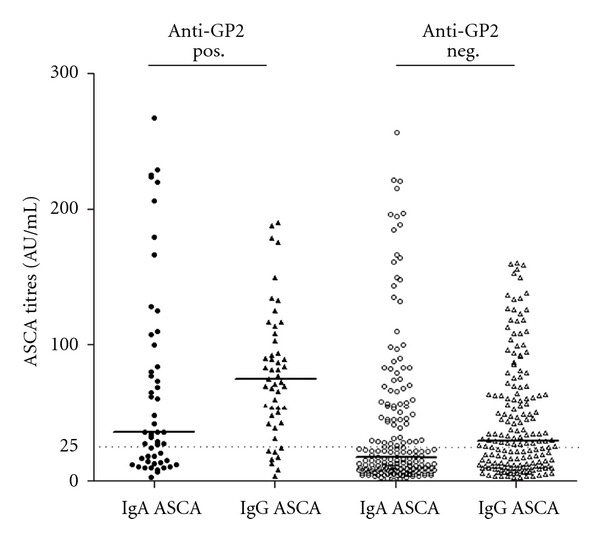
Comparison of IgA and IgG ASCA titres in IgG anti-GP2 antibody positive and negative patients with Crohn's disease (CD). Statistical analysis did not reveal significant differences amongst anti-GP2 antibody positive and anti-GP2 antibody negative CD patients in ASCA titres.

Although, there was no correlation between ASCA (IgA or IgG) and anti-GP2 titres in double positive patients, the titres of ASCA (IgA and IgG) were higher in patients positive for IgG anti-GP2 as shown in Figure 4.

There was no statistically significant difference in the prevalence of ASCA IgA in patients with different locations (L1+L3: 83/180 versus L2: 18/45, *P* = 0.5056). Patients with colonic disease (L2) though had lower prevalence of ASCA IgG (L1+L3: 123/180 versus L2: 19/45,  *P* < 0.0009). 

No difference was found in the prevalence of ASCA IgA in patients with different behaviour phenotypes (B1: 42/108, B2: 33/62 and B3: 24/57) but ASCA IgG were more prevalent in patients with stricturing disease (B1: 58/108, B2: 48/62, and B3: 35/57; *P* = 0.0131).

### 3.4. IgG Anti-GP2 Antibody Association with Other Clinical Parameters

**Figure 5:**
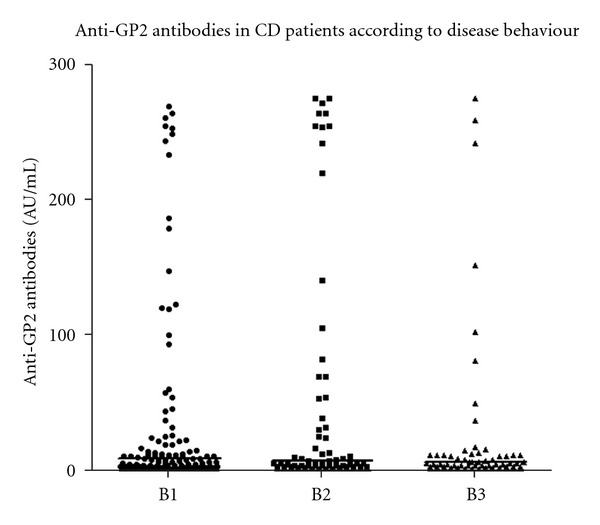
Comparison of IgG anti-GP2 antibodies in patients with Crohn's disease (CD) stratified in accordance to disease behaviour (Montreal Classification, B1, B2, and B3).

There was no statistically significant difference between anti-GP2 IgG positive and negative CD patients in regards to age of disease onset and duration of disease (Figure 4). There was no correlation between anti-GP2 IgG titres and disease duration. The prevalence of IgG anti-GP2 antibodies differed when CD patients were stratified in subgroups according to their disease behaviour (Figure 5), but there was no difference in the titre medians between subgroups. IgG anti-GP2 antibodies were present in 29/106 (27%) CD patients with B1, 22/62 (35%) with B2, and 8/57 (14%) with B3 phenotype (*P* = 0.0046).

 Family history of IBD was not associated with anti-GP2 antibody seropositivity. Among the 57 (25.3%) CD patients with family history of IBD, 13 (22.8%) were positive for anti-GP2 IgG compared to 43 out of 168 (25.6%) without family history of IBD (*P* > 0.05). 

Amongst the 128 CD patients with previous surgical intervention, 45 (35.2%) were positive for anti-GP2 IgG compared to only 14/97 (14.4%) CD patients without surgical history (*χ* ^2^ = 12.25, df = 1, *P* < 0.000, odds ratio: 3.214, 95% CI 1.641 to 6.298).

The prevalence of anti-GP2 antibodies did not differ amongst naive patients or patients at early stages of their disease (duration of disease less than two years) compared to patients with >2 years disease duration (4/22, 18% versus 54/203, 26%, *P* > 0.05). Also, the prevalence of anti-GP2 antibody reactivity did not differ amongst patients treated with or without infliximab (11/38 and 28.9% versus 48/187 and 25.7%; *P* > 0.05).

### 3.5. Behaviour of Anti-GP2 Antibodies over Time

**Figure 6:**
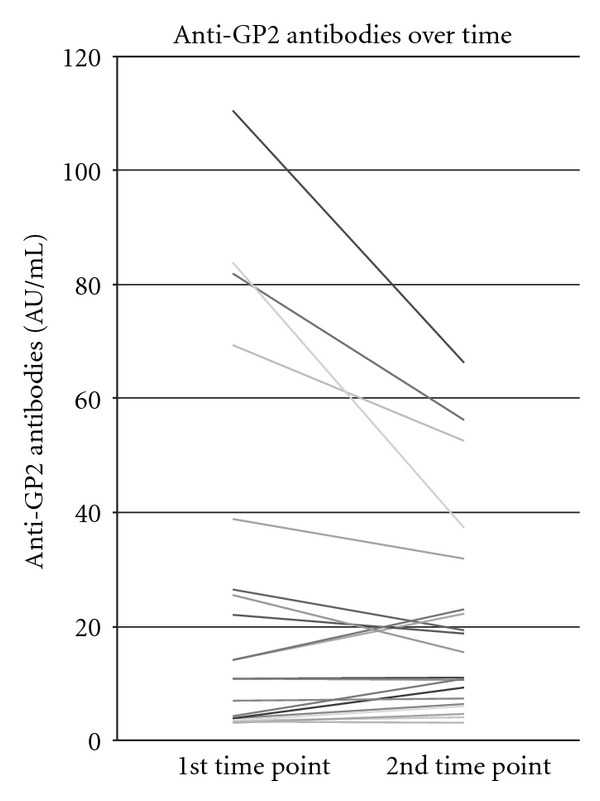
Behaviour of anti-GP2 antibodies during follow up in 20 randomly selected patients with Crohn's disease (CD), including 8 anti-GP2 antibody positive at baseline.

An additional set of experiments was carried out to test the behaviour of anti-GP2 antibodies over time in 20 CD patients, including 8 (20%) who were anti-GP2 antibody positive at baseline. All 8 anti-GP2 antibody positive patients have shown a decline of their GP2 autoantibody titres at repeated testing. Of those, 5 retained their seropositivity and 3 became seronegative. Of the 12 anti-GP2 antibody negative cases at baseline, 2 became seropositive at relatively low titres during follow up (Figure 6). The single UC patient who was positive on initial sampling remained positive on the second test. 

## 4. Discussion

Pancreatic antibodies directed against GP2 have been considered serological markers of CD, being present in approximately 20–36% of patients with IBD [12, 17–19, 21–23]. Why GP2 becomes an autoantigenic target in CD is unclear [12, 13, 24]. Also, why some but not all patients with CD develop humoral autoreactivity against this pancreatic autoantigen remains elusive [12]. Moreover, it is not clear whether these autoantibodies are secondary to intestinal destruction or participate in the induction of the disease.

In the present study, we assumed that patients showing a disease location restricted to the colon, and therefore without inflammation of the ileum, would lack antibody reactivity to GP2 [12]. We based our hypothesis on recent evidence indicating that there is GP2 expression in the intestine in addition to its former known pancreatic site of synthesis. It seems to be limited to the intestinal M cells [11]—the atypical epithelial cells that account for up to 10% of FAE [14, 25, 26]. The role of M cells is generally to phagocytose macromolecules and microbes and to transport them to the underlying mucosa-associated immune system for antigen presentation. Thus, M cells play a crucial role in maintaining the critical balance in terms of recognising and differentiating self and nonself. Intriguingly, M cells and Peyer's patches are particularly abundant in the distal part of the ileum [11, 27], which has been considered to be the most likely site of inflammation onset in newly diagnosed, adolescent patients with CD and is generally one of the most common sites for clinically apparent disease activity [28]. According to the above argument, intestinal inflammation sparing the ileum would not be able to release GP2 from the inflamed tissue. The release of GP2 would be a prerequisite for the activation of the immune system and the initiation of an autoimmune reaction that could lead to the induction of anti-GP2 antibodies [12]. 

Indeed, our study clearly demonstrates that patients with the restricted colonic form of the disease do not show significant antibody reactivity against GP2 compared to those with a disease location that involves the ileum, the site of GP2-rich M cells. The necessity for ileal inflammation in order for anti-GP2 antibodies to be developed can also explain why only a minority of patients with UC have shown autoantibody reactivity against this antigen [12, 17–19, 21–23]. In our study, fewer than 7% of 225 patients with UC have shown anti-GP2 antibodies. This percentage is in agreement with most studies reporting on the prevalence of anti-GP2 antibodies in smaller cohorts of UC patients [8, 17–19]. 

A previous study reporting coexistence of ASCA and anti-GP2 in a significant proportion of CD patients [17] has been followed by other studies that were unable to replicate this finding [21, 22]. Also, comparison of various demographic and clinical parameters analysed in our cohorts was unable to show significant differences in terms of age of disease onset, as well as disease duration, in accordance with previous studies [21]. A lower prevalence was found in patients with penetrating disease B3 and this needs further investigation. Anti-GP2 antibodies were more prevalent in patients with previous surgical intervention than in those without (35% versus 14%, *P* < 0.001). The clinical relevance of this finding will remain uncertain until it is replicated in larger studies. Most studies conducted so far have been unable to provide a decisive outcome, and thus our data need to be interpreted with caution [8, 12, 17, 18, 21–23].

Our study cannot estimate accurately the prevalence of anti-GP2 in patients with CD, and this needs to be noted. Our cohort has an overrepresentation of patients with ileal involvement. Also, most serum samples originate from patients already treated. For an accurate estimation of the prevalence of anti-GP2 antibodies, a large cohort of naïve patients with Crohn's disease has to be tested. Taking into account that over the course of the disease, anti-GP2 antibodies decline, it would be logical to assume that the real prevalence of anti-GP2 antibodies may be higher than that so far reported in cohorts involving sera from already treated patients. Safer conclusions can only be reached if serial, large number of samples collected prospectively over a long-duration of follow up could be tested.

A few other points need to be made. The first point considers whether anti-GP2 antibodies contribute to the development of the disease or whether their existence is just an epiphenomenon following intestinal epithelial destruction [12]. Furthermore, it has been demonstrated that GP2 recognises FimH, which is a constituent of the type 1 pilus expressed on the outer membrane of some enterobacilli, such as *E. coli* and *Salmonella enterica *[11]. FimH have a lectin-like capability to bind certain glycoproteins in a mannose-dependent manner, and appear to be able to recognise GP2 [11, 13]. Specific interaction with FimH positive bacteria found in some but not all CD patients might explain the limited prevalence of anti-GP2 antibodies in IBD patients. Work on IBD animal models and GP2-deficient mice may shed a light on the pathogenic potential of GP2-specific immune responses. The fact that GP2 is expressed on the apical surface of M cells [11, 13] making it accessible to antibodies is of special interest, as it would support the notion that these cells may be the targets of antibody-dependent cytotoxicity [12]. On the other hand, a high proportion of individuals with CD appear seronegative for anti-GP2 antibodies, not only at baseline but also over time. This finding clearly indicates that the loss of immunological tolerance to this antigen is not an *a priori* condition for the development of the disease. There is no doubt that the pathogenesis of CD involves mechanisms other than those responsible for the induction of GP2 autoimmunity [29]. These mechanisms may be important for the establishment of the disease, acting in isolation or in combination with those leading to M cell-related induction of anti-GP2 antibodies, seen in over a quarter of patients with Crohn's disease. Why only those and not all patients develop these autoantibodies remains unclear. As anti-GP2 is absent in approximately 74% of patients with CD, the validity of the isolated detection of GP2-specific PAB is impaired. Most investigators agree that the routine use of isolated serological markers for diagnosis and especially for the follow up of patients with inflammatory diseases is limited by their inadequate performance in terms of the diagnosis and prognosis of CD [29–31]. ASCA, for example, are more prevalent in patients with CD and their participation in the routine testing of patients with CD is more than adequate. In conclusion, we share with others the notion that several serological markers must be used in combination to be more effective compared to isolated/single marker testing.

Nevertheless, anti-GP2 antibody testing appears to be one of those tests that can be added in the diagnostic workup of patients with CD. More work needs to be done over the next few years to understand the immunobiological role of this antigen and its relevance to IBD.

Emerging data indicating an important immunoregulatory role of GP2 for the emergence of innate and adaptive immunity (including the recruitment of regulatory T cells) in the intestine [32] may initiate an intense research in this field and elucidate the role of this interesting autoantigen. 

## Figures and Tables

**Table 1:** Main demographic and clinical characteristics of the 225 patients with Crohn's disease (CD) and the 225 patients with ulcerative colitis (UC) included in the present study.

	CD	UC
*N*	225	225
Sex (m/f)	98 (43.6%)/127 (56.4%)	113 (50.2%)/112 (49.8%)
Age (mean ± SD)	36 ± 14.3	51 ± 15.7
Age at diagnosis (mean ± SD)	23 ± 11.6	30 ± 14.6
Disease duration (mean ± SD)	13 ± 10.1	14 ± 12.9
Location *n* (%)	L1: 45 (20%)	E1: 28 (12.4%)
	L2: 45 (20%)	E2: 66 (29.3%)
	L3: 135 (60%)	E3: 131 (58.2%)
Behaviour *n* (%)	B1: 106 (47%)	
	B2: 62 (28%)	
	B3: 57 (25%)	
	Perianal: 60 (27%)	
Age	A1: 46 (20%)	
	A2: 156 (70%)	
	A3: 23 (10%)	
